# COVID-19 Outcomes in Myasthenia Gravis Patients: Analysis From Electronic Health Records in the United States

**DOI:** 10.3389/fneur.2022.802559

**Published:** 2022-03-28

**Authors:** Youngran Kim, Xiaojin Li, Yan Huang, Minseon Kim, Aziz Shaibani, Kazim Sheikh, Guo-Qiang Zhang, Thy Phuong Nguyen

**Affiliations:** ^1^Department of Neurology, McGovern Medical School, The University of Texas Health Science Center at Houston, Houston, TX, United States; ^2^Nerve and Muscle Center of Texas, Houston, TX, United States

**Keywords:** COVID-19, myasthenia gravis, mortality, rheumatoid arthritis, systemic lupus, multiple sclerosis, electronic medical records, coronavirus

## Abstract

**Background:**

Myasthenia gravis (MG) is an autoimmune, neuromuscular condition and patients with MG are vulnerable due to immunosuppressant use and disease manifestations of dyspnea and dysphagia during the coronavirus disease 2019 (COVID-19) pandemic.

**Methods:**

We conducted a retrospective cohort study using the Optum^®^ de-identified COVID-19 Electronic Health Record (EHR) dataset. Primary outcomes, such as hospitalization, ventilator use, intensive care unit (ICU) admission, and death in COVID-19 patients with MG, were compared with those of COVID-19 patients without MG: the subgroups of non-MG included those with rheumatoid arthritis (RA), systemic lupus (SLE), and multiple sclerosis (MS). We further analyzed factors affecting mortality, such as age, race/ethnicity, comorbidities, and MG treatments.

**Results:**

Among 421,086 individuals with COVID-19, there were 377 patients with MG, 7,362 patients with RA, 1,323 patients with SLE, 1,518 patients with MS, and 410,506 patients without MG. Patients with MG were older and had more comorbidities compared with non-MG patients and had the highest rates of hospitalization (38.5%), ICU admission (12.7%), ventilator use (3.7%), and mortality (10.6%) compared with all other groups. After adjusting for risk factors, patients with MG had increased risks for hospitalization and ICU compared with patients with non-MG and with RA but had risks similar to patients with SLE and with MS. The adjusted risk for ventilator use was similar across all groups, but the risk for mortality in patients with MG was lower compared with the SLE and MS groups. Among patients with MG, age over 75 years and dysphagia were predictors for increased COVID-19 mortality, but the recent MG treatment was not associated with COVID-19 mortality.

**Conclusions:**

COVID-19 patients with MG are more likely to be admitted to the hospital and require ICU care. Older age and patients with dysphagia had an increased risk of mortality.

## Introduction

The coronavirus disease 2019 (COVID-19) pandemic has fundamentally altered neurologic care in several ways. First, there is a growing body of evidence to suggest that COVID-19 can cause neurological manifestations directly by the infection or by the body's innate and adaptive immune responses to the infection (1–3). Additionally, patients with underlying or baseline neurologic diseases can be impacted by the COVID-19 pandemic, whether they are infected or not (1). Many neurological patients with multiple sclerosis (MS) or autoimmune diseases, such as myasthenia gravis (MG), are often treated with immunosuppressive medications and are likely to have significant comorbidities that may be directly related to their treatment (1). The COVID 19 pandemic may be associated with delayed or suspended initiation of treatment, maintenance of treatment, and re-initiation of treatment after the COVID-19 infection and may also impact the complications of treatment and hospitalized patient outcomes. COVID-19 generates multiple management issues, particularly in patients with autoimmune diseases, such as MG.

Myasthenia gravis is the most common autoimmune condition affecting the neuromuscular junction (4). Patients with MG are uniquely vulnerable to respiratory infections given their underlying neuromuscular weakness, leading to dyspnea and dysphagia (5). Additionally, effective MG drugs often lead to immunosuppression, which could both predispose and alter the response to virus treatments (6–9). Therefore, patients with MG may be at increased risk for severe COVID-19 caused by severe acute respiratory syndrome coronavirus-2 (SARS-CoV-2) (10, 11). Treating neurologists are called to guide patients on decisions regarding the maintenance of current treatment, initiation of new treatments, prevention, and vaccination during this global pandemic (5, 6, 12, 13). However, effective counseling for patients is dependent on the knowledge of outcomes and the impact of COVID-19 on MG. The current literature consists primarily of case reports, small case series, expert consensus, and a single physician-reported registry with the reports of the interim analysis (6, 10, 11, 14–23). Previously reported mortality rates in COVID-19 with MG range from 6.8 to 30%. Additionally, prior studies reported increased risks for hospitalization and death in COVID-19 patients with MG compared with those without MG (10, 11, 15). COVID-19 mortality rates in MG are significantly higher than the known mortality of MG hospitalization: 2.2% for overall in-hospital mortality and 4.47% for MG crisis (24). We aimed to evaluate hospitalization, intensive care unit (ICU) admission, ventilator use, and all-cause death in COVID-19 among patients with MG and compare these outcomes with non-MG and other disease groups that shared treatments or disease characteristics with MG. In addition, we determined factors associated with poor outcomes in COVID-19 patients with MG.

## Methods

### Data Source

We conducted a retrospective cohort study using the Optum^®^ de-identified COVID-19 Electronic Health Record (EHR) dataset (25). The COVID-19 data are sourced from Optum^®^'s longitudinal EHR repository derived from more than 700 hospitals and 7,000 clinics in the United States with a minimal time lag while preserving as much clinical information as possible. The most recent data we used at the time of the study included 4.2 million unique individuals and covered until March 31, 2021. The study was reviewed and approved by the Committee for the Protection of Human Subjects (CPHS) at The University of Texas Health Science Center at Houston and followed the Strengthening the Reporting of Observational Studies in Epidemiology (STROBE) reporting guideline.

### Study Population

**Figure 1:**
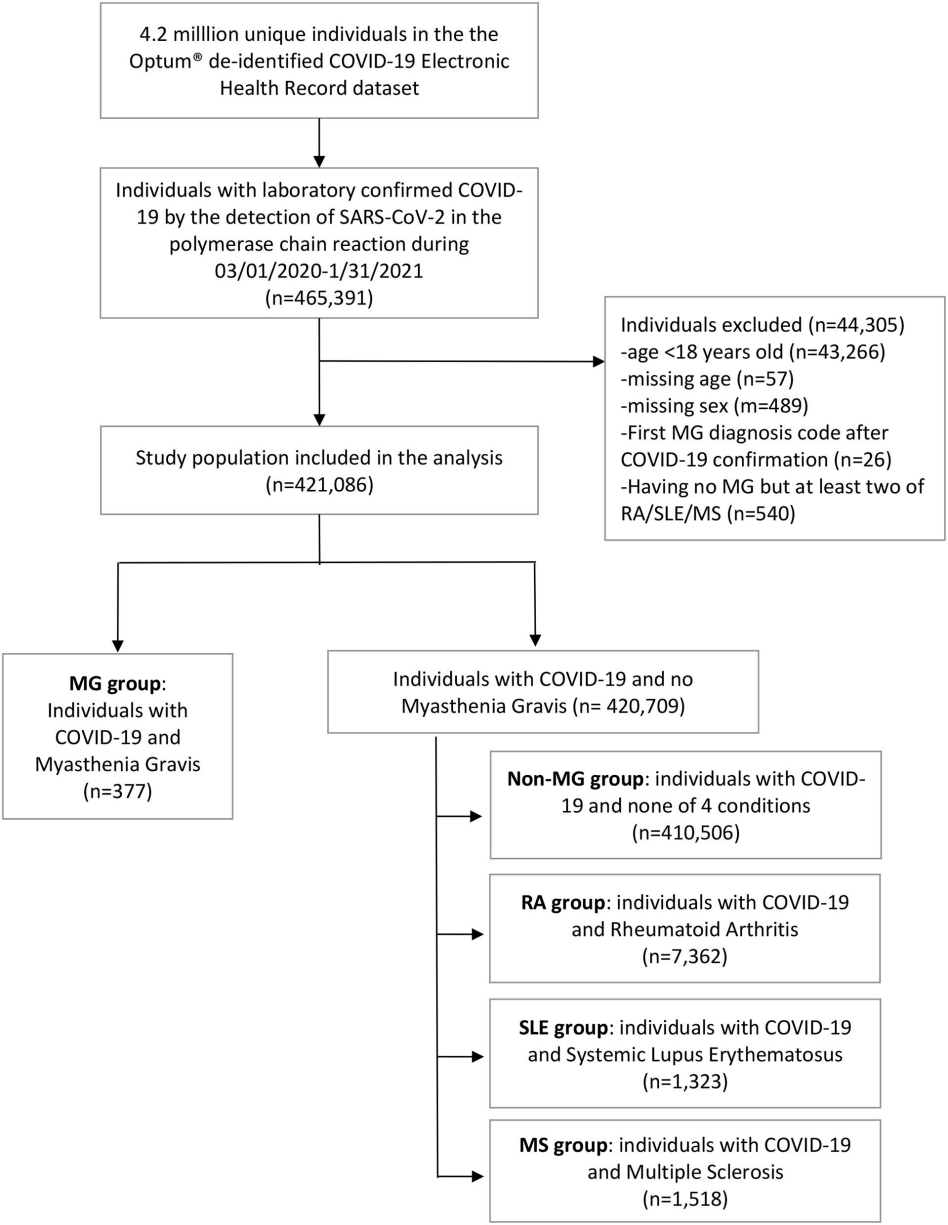
Cohort derivation.

Patients were included if they had laboratory-confirmed COVID-19 by the detection of SARS-CoV-2 in the polymerase chain reaction (PCR) test between March 1, 2020 and January 31, 2021 (*n* = 465,391). Patients who were younger than 18 years old (*n* = 43,266), with unknown age (*n* = 57), unknown sex information (*n* = 489), or the first record of MG diagnosis after COVID-19 confirmation (*n* = 26) were excluded (Figure 1). MG was determined using the International Classification of Diseases, Ninth Revision, Clinical Modification (ICD-9-CM) 358.0x or ICD-10-CM G70.0x. Among COVID-19 patients without MG, we further identified patients with rheumatoid arthritis (RA), systemic lupus erythematosus (SLE), and multiple sclerosis (MS) based on ICD-9 and 10-CM codes (Supplementary Table 1). If patients had multiple conditions of RA, SLE, or MS among the non-MG group, they were excluded (*n* = 540). Patients with these conditions were categorized as RA, SLE, MS groups, and the remaining non-MG patients without the diagnoses of RA, SLE, and MS were categorized as a non-MG group. We selected RA and SLE as comparison groups because we wanted to determine if there were unique vulnerabilities of the MG patient population to COVID-19 in comparison to other autoimmune diseases that may have similar treatments. We selected MS as another comparison group to determine if COVID-19 outcomes differed compared with another autoimmune neurologic disease affecting the central nervous system. Although ICD-9-CM codes have been replaced with ICD-10-CM as of October 2015, ICD-9 codes were included to identify MG, RA, SLE, MS, and other comorbidities as some patient medical records were still reported using ICD-9 codes.

### Measurements

Our primary objective was to determine the effect of MG on the outcomes of COVID-19, such as hospitalization, ICU admission, ventilator use, and all-cause deaths occurring within 45 days from COVID-19 diagnosis. COVID-19 outcomes were compared across disease groups adjusting for baseline characteristics. The secondary objective was to identify factors associated with mortality in COVID-19 patients with MG. Potential predictors included age, sex, race/ethnicity, comorbidities, dysphagia, dyspnea, and recent MG treatments within 6 months before the COVID-19 diagnosis. Comorbidities included chronic pulmonary disease, cardiovascular disease, cerebrovascular disease, peripheral vascular disease, diabetes, liver disease, renal disease, obesity, and smoking that were identified using ICD-9 and 10 CM codes (Supplementary Table 1). MG treatments were categorized as symptomatic treatment (acetylcholinesterase inhibition and pyridostigmine), chronic immunosuppressive therapies (prednisone, azathioprine, or mycophenolate), and intravenous immune globulin (IVIG).

### Statistical Analysis

Descriptive statistics for differences in characteristics between the MG and the non-MG groups were assessed using the chi-square tests for categorical variables and the Wilcoxon rank-sum tests for numeric variables. The COVID-19 outcomes were compared across disease groups, and risk ratios (*RR*s) were estimated using the modified multivariable Poisson regression models (26) adjusted for age, sex, race/ethnicity, region, comorbidities, and COVID-19 test month. The month of diagnosis of COVID-19 was used as an adjusting variable as mortality improved with later diagnosis during the pandemic. As a sensitivity analysis, we repeated analysis excluding MG patients with RA, SLE, or MG. The associations between potential predictors, such as age, sex, race/ethnicity, region, COVID-19 test month, comorbidities, dysphagia, dyspnea, and recent MG treatments, and mortality in an MG group were measured as *RR*s from a modified multivariable Poisson regression model. Significance levels were set at *p* < 0.05 for 2-tailed tests, and all analyses were performed using STATA 16.0 (StataCorp, College Station, TX).

## Results

### Baseline Characteristics of Patients With COVID-19

A total of 421,086 individuals with COVID-19 were included. There were 377 individuals in the MG group, 7,362 in the RA group, 1,323 in the SLE group, 1,518 in the MS group, and 410,506 in the non-MG group without any of 4 conditions (Figure 1). Individuals in the MG group compared with those in the non-MG group were older (median age 68 vs. 47 years, *p* < 0.001), more likely to be white (78 vs. 65%, *p* < 0.001), and had 2–3 times higher prevalence of comorbidities (Table 1). Chronic pulmonary disease, cardiovascular disease, diabetes, dysphagia, dyspnea, and obesity were very common in the MG group (Table 1). Among patients with MG, 14% were with co-existent RA (8%), SLE (3%), or MS (5%). Individuals in the RA, SLE, and MS groups were younger and had a much higher percentage of women compared with those in the MG group. Among individuals in the MG group, 59% were on chronic immunosuppressive therapies (55% on prednisone, 9% on azathioprine, and 11% on mycophenolate), 37% on symptomatic treatment (acetylcholinesterase inhibition and pyridostigmine), and 8% were recently treated with IVIG within 6 months before the COVID-19 diagnosis.

**Table 1:** The characteristics of patients with coronavirus disease 2019 (COVID-19)^*^.

	**Non-MG** **(*n* = 410,506)**	**MG** **(*n* = 377)**	**RA ** **(*n* = 7,362)**	**SLE ** **(*n* = 1,323)**	**MS ** **(*n* = 1,518)**
**Age, median (IQR)**	47 (32–61)	68 [56–77]	63 [52–73]	51 [40–62]	55 [43–64]
**Male**, ***n*** **(%)**	186,088 (45.3)	186 (49.3)	2,104 (28.6)	162 (12.2)	369 (24.3)
**Race/Ethnicity**, ***n*** **(%)**					
White	268,338 (65.4)	292 (77.5)	5,474 (74.4)	769 (58.1)	1,154 (76.0)
Black	46,341 (11.3)	40 (10.6)	890 (12.1)	295 (22.3)	193 (12.7)
Hispanic	47,752 (11.6)	23 (6.1)	638 (8.7)	186 (14.1)	95 (6.3)
Other/unknown	48,075 (11.7)	22 (5.8)	360 (4.9)	73 (5.5)	76 (5.0)
**Region**, ***n*** **(%)**					
Northeast	81,903 (20.0)	64 (17.0)	1,511 (20.5)	269 (20.3)	374 (24.6)
Midwest	212,270 (51.7)	200 (53.1)	3,619 (49.2)	597 (45.1)	748 (49.3)
South	76,790 (18.7)	95 (25.2)	1,712 (23.3)	323 (24.4)	233 (15.3)
West	25,198 (6.1)	10 (2.7)	356 (4.8)	84 (6.3)	120 (7.9)
Other/Unknown	14,345 (3.5)	8 (2.1)	164 (2.2)	50 (3.8)	43 (2.8)
**Comorbidities**, ***n*** **(%)**					
Chronic pulmonary disease	97,338 (23.7)	189 (50.1)	3,636 (49.4)	631 (47.7)	527 (34.7)
Cardiovascular disease	88,972 (21.7)	236 (62.6)	3,722 (50.6)	551 (41.6)	530 (34.9)
Cerebrovascular disease	28,345 (6.9)	113 (30.0)	1,400 (19.0)	221 (16.7)	261 (17.2)
Peripheral vascular disease	27,883 (6.8)	94 (24.9)	1,605 (21.8)	200 (15.1)	193 (12.7)
Diabetes	73,832 (18.0)	162 (43.0)	2,645 (35.9)	379 (28.6)	332 (21.9)
Liver disease	31,897 (7.8)	70 (18.6)	1,397 (19.0)	228 (17.2)	188 (12.4)
Renal disease	35,615 (8.7)	117 (31.0)	1,772 (24.1)	327 (24.7)	198 (13.0)
Hemiplegia or paraplegia	5,821 (1.4)	29 (7.7)	266 (3.6)	56 (4.2)	229 (15.1)
Dysphagia	25,557 (6.2)	148 (39.3)	1,250 (17.0)	217 (16.4)	296 (19.5)
Dyspnea	22,671 (5.5)	78 (20.7)	945 (12.8)	203 (15.3)	129 (8.5)
Obesity	127,345 (31.0)	205 (54.4)	3,850 (52.3)	633 (47.8)	610 (40.2)
Smoking	52,335 (12.7)	53 (14.1)	1,354 (18.4)	227 (17.2)	295 (19.4)
**MG treatment**, ***n*** **(%)**					
Prednisone	NA	207 (54.9)	3,387 (46.0)	602 (45.5)	537 (35.4)
Pyridostigmine	NA	138 (36.6)	2 (0.0)	0 (0.0)	2 (0.1)
IVIG	NA	30 (8.0)	30 (0.4)	12 (0.9)	6 (0.4)
Azathioprine	NA	33 (8.8)	47 (0.6)	43 (3.3)	4 (0.3)
Mycophenolate	NA	42 (11.1)	47 (0.6)	114 (8.6)	9 (0.6)

*MG, myasthenia gravis; RA, rheumatoid arthritis; SLE, systemic lupus erythematosus; MS, multiple sclerosis; IQR, interquartile range; IVIG, intravenous immune globulin*.

* *Differences in characteristics between the non-MG and MG groups were assessed using the chi-square tests for categorical variables and the Wilcoxon rank-sum tests for numeric variables and were statistically significant (p < 0.001) except for sex (p = 0.12), and smoking status (p = 0.45)*.

### COVID-19 Outcomes

More than one-third (38.5%) of MG patients with COVID-19 were hospitalized as well as 14.0% of non-MG, 25.8% of RA, 23.8% of SLE, and 23.6% of MS patients with COVID-19 were hospitalized (Table 1). After adjusting for the covariates of age, sex, race/ethnicity, region, month of COVID-19 test, and comorbid conditions, patients with MG, SLE, and MS all had more than 20% increased risk for hospitalization compared with those with non-MG, while patients with RA had a similar risk for hospitalization compared with those with non-MG: 28% higher in the MG group (adjusted *RR* [a*RR*] 1.28, 95% *CI* 1.13–1.46, *P* < 0.001), 20% higher (a*RR* 1.20, 95% *CI* 1.10–1.31, *p* < 0.001) in the SLE group, and 39% higher (a*RR* 1.39, 95% *CI* 1.27–1.51, *p* < 0.001) in the MS group compared with the non-MG group (Table 2). Compared to patients with MG, patients with RA had a 22% (a*RR* 0.78, 95% *CI* 0.69–0.89, *p* < 0.001) lower risk of hospitalization, while patients with SLE and with MS had similar risks of hospitalization. ICU admission (12.7%) in patients with MG was 4 times higher compared with the non-MG group and 2 times higher compared with the RA, SLE, MS groups. After consideration of covariates, the risk for ICU admission was 51% higher in patients with MG (a*RR* 1.51, 95% *CI* 1.16–1.96, *p* = 0.002) compared with patients with non-MG and was similar to those in patients with SLE and with MS. Ventilator use (3.7%) was higher in the MG group compared with non-MG or other groups, but differences became insignificant after the covariate analysis. Mortality rates were three times higher in patients with MG (10.6%) and more than two times higher in patients with RA (7.0%) compared with patients in the non-MG group (3.0%). However, after covariate analysis, these differences were no longer significant. Also, after covariate adjustment, mortality rates in MG were similar to the RA group. Patients with SLE (5.7%) and with MS (5.2%) had higher unadjusted mortality than patients in the non-MG group (3.0%) but had them lower than patients with MG (10.6%) and with RA (7.0%). After covariate analysis, risks were 48% (a*RR* 1.48, 95% *CI* 1.04–2.10, *p* = 0.020) and 42% (a*RR* 1.42, 95% *CI*, 1.01–2.01, *p* = 0.036), respectively, higher than patients with MG. When we compared COVID-19 outcomes between MG only vs. MG with other conditions, we found no significant differences between the two groups and results remained similar if we excluded those with co-existent conditions (Supplementary Table 2).

**Table 2:** The comparisons of COVID-19 outcomes.

	**No. (%)**	**Crude RR (95% CI)**	***p*-value**	Adjusted RR^*^in reference to None (95% CI)	***p*-value**	Adjusted RR^*^in reference to MG (95% CI)	***p*-value**
**Hospitalization**							
None	57,613 (14.0)	1.00 (Reference)		1.00 (Reference)		0.78 (0.69–0.89)	<0.001
MG	145 (38.5)	2.74 (2.41–3.11)	<0.001	1.28 (1.13–1.46)	<0.001	1.00 (Reference)	
RA	1,903 (25.8)	1.84 (1.77–1.92)	<0.001	1.01 (0.97–1.04)	0.78	0.78 (0.69–0.89)	<0.001
SLE	315 (23.8)	1.70 (1.54–1.87)	<0.001	1.20 (1.10–1.31)	<0.001	0.94 (0.80–1.09)	0.41
MS	358 (23.6)	1.68 (1.53–1.84)	<0.001	1.39 (1.27–1.51)	<0.001	1.08 (0.93–1.26)	0.31
**ICU**							
None	13,561 (3.3)	1.00 (Reference)		1.00 (Reference)		0.66 (0.51–0.86)	0.002
MG	48 (12.7)	3.85 (2.96–5.02)	<0.001	1.51 (1.16–1.96)	0.002	1.00 (Reference)	
RA	503 (6.8)	2.07 (1.90–2.25)	<0.001	1.00 (0.92–1.09)	0.92	0.67 (0.51–0.88)	0.004
SLE	79 (6.0)	1.81 (1.46–2.24)	<0.001	1.18 (0.96–1.46)	0.11	0.79 (0.56–1.10)	0.16
MS	92 (6.1)	1.83 (1.50–2.24)	<0.001	1.43 (1.18–1.74)	<0.001	0.95 (0.69–1.32)	0.77
**Ventilator**							
None	4,892 (1.2)	1.00 (Reference)		1.00 (Reference)		0.87 (0.52–1.46)	0.59
MG	14 (3.7)	3.12 (1.86–5.21)	<0.001	1.15 (0.69–1.93)	0.60	1.00 (Reference)	
RA	161 (2.2)	1.84 (1.57–2.14)	<0.001	0.92 (0.78–1.07)	0.27	0.80 (0.47–1.36)	0.41
SLE	32 (2.4)	2.03 (1.44–2.86)	<0.001	1.30 (0.92–1.83)	0.13	1.13 (0.61–2.10)	0.70
MS	33 (2.2)	1.82 (1.30–2.56)	0.001	1.30 (0.94–1.81)	0.12	1.13 (0.62–2.09)	0.69
**Death**							
None	12,211 (3.0)	1.00 (Reference)		1.00 (Reference)		0.92 (0.69–1.23)	0.59
MG	40 (10.6)	3.57 (2.66–4.78)	<0.001	1.08 (0.81–1.44)	0.59	1.00 (Reference)	
RA	518 (7.0)	2.37 (2.17–2.57)	<0.001	1.02 (0.94–1.10)	0.67	0.94 (0.70–1.26)	0.69
SLE	75 (5.7)	1.91 (1.53–2.38)	<0.001	1.60 (1.30–1.97)	<0.001	1.48 (1.04–2.10)	0.020
MS	79 (5.2)	1.75 (1.41–2.17)	<0.001	1.54 (1.26–1.88)	<0.001	1.42 (1.01–2.01)	0.036

*RR, risk ratio; MG, myasthenia gravis; RA, rheumatoid arthritis; SLE, systemic lupus erythematosus; MS, multiple sclerosis*.

* *The COVID-19 outcomes were compared across disease groups and risk ratios (RRs) were estimated using modified multivariable Poisson regression models adjusting for age, sex, race/ethnicity, region, COVID-19 test month, and comorbidities, such as chronic pulmonary disease, cardiovascular disease, cerebrovascular disease, peripheral vascular disease, diabetes, liver disease, renal disease, obesity, and smoking*.

### Factors Associated With Increased Mortality in the MG Group

Among COVID-19 patients with MG, ages 65–74 and 75 years or older were associated with an increased risk of death, but only age 75 years or older remained significant after adjusting for covariates (Table 3). Male gender and Black/Hispanic race/ethnicitywere associated with increased risk for death but they were not statistically significant. Cardiovascular disease, peripheral vascular disease, and dysphagia were significantly associated with an increased risk of death in the univariate analysis, but only dysphagia remained significant after multivariable analysis. We found no association between recent MG treatments and mortality in COVID-19 with MG.

**Table 3:** Factors associated with mortality in COVID-19 with myasthenia gravis (MG).

	**Crude RR (95% CI)**	***p*-value**	Adjusted RR^*^(95% CI)	***p*-value**
**Age**				
18–64	1.00 (Reference)		1.00 (Reference)	
65–74	5.50 (1.57–19.27)	0.008	4.95 (0.78–31.62)	0.09
75+	11.56 (3.58–37.32)	<0.001	9.57 (1.56–58.76)	0.015
**Sex**				
Female	1.00 (Reference)		1.00 (Reference)	
Male	1.39 (0.77–2.52)	0.28	1.35 (0.74–2.46)	0.32
**Race/Ethnicity**				
White	1.00 (Reference)		1.00 (Reference)	
Black	0.42 (0.10–1.67)	0.22	1.66 (0.22–12.67)	0.62
Hispanic	0.73 (0.19–2.83)	0.64	1.95 (0.54–7.03)	0.31
Other/unknown	0.38 (0.05–2.65)	0.33	0.43 (0.05–3.97)	0.46
**Comorbidities**				
Chronic pulmonary disease	1.66 (0.90–3.05)	0.10	1.27 (0.64–2.51)	0.49
Cardiovascular disease	5.38 (1.95–14.81)	0.001	2.62 (0.99–6.96)	0.05
Cerebrovascular disease	1.40 (0.77–2.56)	0.27	1.04 (0.54–2.04)	0.90
Peripheral vascular disease	2.46 (1.38–4.39)	0.002	1.53 (0.72–3.25)	0.27
Diabetes	1.09 (0.60–1.96)	0.78	0.85 (0.49–1.48)	0.57
Liver disease	0.63 (0.25–1.54)	0.31	0.65 (0.24–1.76)	0.40
Renal disease	1.48 (0.82–2.68)	0.20	0.70 (0.38–1.28)	0.24
Dysphagia	2.09 (1.16–3.79)	0.015	1.84 (1.06–3.21)	0.031
Dyspnea	1.11 (0.55–2.24)	0.300	0.71 (0.33–1.49)	0.36
**Recent MG treatment**				
Pyridostigmine	1.28 (0.71–2.31)	0.41	0.96 (0.47–1.98)	0.91
Chronic immunosuppressants	1.59 (0.84–3.04)	0.16	1.27 (0.61–2.63)	0.52
IVIG	0.94 (0.31–2.87)	0.91	0.79 (0.15–4.03)	0.77

*RR, risk ratio; MG, myasthenia gravis; IVIG, intravenous immune globulin*.

* *Adjusted RR (aRR) were reported from modified Poisson regression including age, sex, race/ethnicity, region, COVID-19 test month, and comorbidities, such as chronic pulmonary disease, cardiovascular disease, cerebrovascular disease, peripheral vascular disease, diabetes, liver disease, renal disease, dysphagia, and dyspnea*.

## Discussion

In this study of a large COVID-19 EHR database, we found that COVID-19 patients with MG had high hospitalization (38.5%), ICU admission (12.7%), ventilator use (3.7%), and mortality (10.6%). After adjusting for covariates, MG was associated with 28% increased hospitalization and 51% increased ICU admission, while it was not statistically associated with an increased risk of ventilator use or mortality. When we compared COVID-19 outcomes in patients with MG with those in other relevant diseases, we found that patients with RA had a lower risk of hospitalization and ICU admission, while those with SLE had an increased risk of death compared with patients with MG. Factors associated with increased mortality with MG-COVID were age 75 years and older and the presence of dysphagia.

We found no evidence that any recent MG treatment was associated with the worsening outcomes of COVID-19 among patients with MG. Having any symptomatic treatment, chronic immunosuppressive therapies, or IVIG within 6 months before the COVID-19 diagnosis was not statistically associated with mortality in COVID-19 patients with MG, adjusting for covariates. A recent retrospective case series of 93 MG patients with COVID-19 found corticosteroid use to be associated with the severity of COVID-19 infection. That study evaluated the different outcomes of COVID-19 severity (severity of pneumonia) compared with our study. Additionally, they obtained data on prednisone dose and the duration of treatment. Although our study evaluated the outcomes of mortality, ICU admission, and hospitalization rather than the COVID-19 severity of infection, many would presume that the COVID-19 severity of infection would have a direct result on our studied outcomes. However, our patients had a significantly higher rate of comorbidities as compared with the Czech case series (27). The rate of comorbidities in our patient population is in line with previous estimates of a high rate of comorbidities in MG (28). This difference in adjustment for a higher rate of comorbidities and covariate analysis may drive the lack of association of worse outcomes with any MG treatment in our study. Multiple other small studies have not shown an unfavorable outcome with the use of immunosuppressants/steroids (14, 15, 17). However, it is feasible that the long-term use of specific MG treatments, such as prednisone, can lead to the development of specific comorbidities, such as obesity, diabetes, and heart disease (28). These comorbidities have been previously shown to worsen the COVID-19 outcomes (29).

The rate of ventilator use was significantly lower than ICU admission in our MG COVID cohort. This may be due to close monitoring of patients with MG in the ICU on Non-invasive positive pressure ventilation (BiPAP). Our estimates of hospitalizations, ICU admissions, and mortality appear to be lower than previous MG registry-based studies (10) but similar to other EHR database studies (11). Registry-based studies may include more severe cases that require hospitalization and medical intervention due to recall bias. The mortality rate of 10.6% in our study is in line with the prior reported mortality values of MG with COVID-19 (6.8–30%) (10, 11, 15). The interim analysis of the CARE-MG registry was published earlier in the pandemic. In our analysis, patients with COVID earlier in the pandemic generally had worse outcomes. Therefore, our inclusion of the 13 months of analysis during the COVID-19 pandemic may also reflect advances in COVID-specific treatment. In addition, our diverse and large sample size allowed us to compare the COVID-19 outcomes between MG and other autoimmune diseases with similar treatments or disabilities and allowed us to explore the MG-specific predictors of COVID-19 outcomes.

Additionally, we identified a small cohort of MG patients with co-existent RA, SLE, and/or MS. Of note, the co-occurrence of multiple autoimmune (AI) diseases can occur in the 5% of patients with AI (30). About 13% of patients with MG based on pooled estimates have co-existent AI disease (31). The most common AI disease co-occurring with MG is autoimmune thyroid disease, followed by SLE and RA (32, 33). Although considered rare, there are the reports of the co-occurrence of MG and MS (31, 34). In our small sample, the rate of co-occurrence of MG and RA and MG and MS is higher than previous reports or pooled estimates (30, 31, 34). This may be due to a higher percentage of the older population in COVID-19 compared with the general MG population and due to limitations of identifying patients on ICD codes and/or misdiagnosis of similar symptoms (MG and MS). The co-occurrence of SLE and MG is consistent with prior reports (33).

This is a limited study as defining MG cohort and the study outcomes of inpatient stay, ICU stay, ventilator use, and death were obtained from the EHS derived database. The validity of ICD codes for MG could not be ascertained due to limited reports of MG antibody values or electrodiagnostic study data. Due to the inability to review patient notes, we were not able to ascertain the percentage of patients with MG who experienced MG exacerbation requiring rescue treatment during the COVID-19 infection. Additionally, data on other immunomodulating treatments used for MG, such as rituximab or eculizumab, or the dose of corticosteroid and duration was not available. Specific parameters of pre-COVID MG status were not available as well, such as MG-ADL scales, forced vital capacity, MG composite, or quantitative MG scale. Given the inability to ascertain MG baseline disease status, precise treatment regimen for MG prior to COVID-19 infection, and MG antibody serotype, it is possible that our cohort included more people with ocular MG or acetylcholine receptor antibody-positive subtype rather than refractory MG subtype (35). In addition to our studied outcomes, the COVID-19 pandemic significantly influenced the quality of life of patients with MG (36). Patients with MG may have more fear of the COVID-19 infection, likely due to their immunosuppressant maintenance treatments. This may have led to an early retirement from work, social isolation, and economic hardships. The fear of COVID-19 infection may have led to behavioral changes that would affect the rate of COVID-19 infections. Although there are several limitations to an EHR-derived database, to our knowledge, this is the largest cohort of MG patients with COVID-19. Our study is unique in that we are able to compare outcomes in MG patients with COVID-19 to multiple groups that may have similar treatments (RA, SLE, and MS), adjusting for multiple covariates known to worsen outcomes in COVID-19.

## Conclusion

In this large cohort of MG patients with PCR-confirmed COVID-19, MG was associated with increased hospitalization and ICU care but was not statistically associated with ventilator use or mortality. COVID-19 patients with MG had a higher risk of hospitalization and ICU admission compared with COVID-19 patients with RA, but a lower risk of death compared to those with SLE. COVID-19 patients with MG aged 75 years or older and underlying dysphagia need to be closely monitored as these factors increased the mortality in patients with COVID-19 MG. We found no evidence that any recent MG treatment was associated with worsening outcomes of COVID-19 among patients with MG. These findings based on real-world data can guide treating neurologists as they continue to counsel their patients on the serious impact of the pandemic on this particular group of patients. This information can guide further decision-making and counseling on vaccination, maintenance treatment, and the initiation of new treatments during the pandemic.

## Data Availability Statement

The data analyzed in this study was obtained from Optum^®^, the following licenses/restrictions apply: Requests to access these datasets must be approved by Optum^®^. Requests to access these datasets should be directed to G-QZ, Guo-Qiang.Zhang@uth.tmc.edu.

## Ethics Statement

The study protocol was reviewed and approved by the Committee for the Protection of Human Subjects (CPHS) at the University of Texas Health Science Center at Houston. Written informed consent for participation was not required for this study in accordance with the national legislation and the institutional requirements.

## Author Contributions

YK and TN designed the study and drafted the manuscript. XL, YK, YH, and G-QZ extracted and curated the data. TN, AS, KS, and MK provided clinical expertise for the interpretation of results. All authors listed have made a substantial, direct, and intellectual contribution to the work and have approved it for publication. All authors contributed to the article and approved the submitted version.

## Conflict of Interest

The authors declare that the research was conducted in the absence of any commercial or financial relationships that could be construed as a potential conflict of interest.

## Publisher's Note

All claims expressed in this article are solely those of the authors and do not necessarily represent those of their affiliated organizations, or those of the publisher, the editors and the reviewers. Any product that may be evaluated in this article, or claim that may be made by its manufacturer, is not guaranteed or endorsed by the publisher.
