# Supplementary material for: COVID-19 Outcomes in Myasthenia Gravis Patients: Analysis From Electronic Health Records in the United States
Source: Front Neurol. 2022 Mar 28;13:802559. doi: 10.3389/fneur.2022.802559 (PMC8996116; doi:10.3389/fneur.2022.802559)
Supplement: Supplementary file 1 [file Data_Sheet_1.PDF]

Supplemental Table 1. International Classification of Diseases (ICD) Codes to Identify Disease Conditions

Conditions	ICD-9-CM	ICD-10-CM
Rheumatoid arthritis	714.x	M05.x, M06.x
Systemic lupus erythematosus	710.0	M32.x
Multiple sclerosis	340	G35
Chronic pulmonary disease	416.8, 416.9, 490.x - 505.x, 506.4, 508.1, 508.8	I27.8x, I27.9, J40.x - J47.x, J60.x - J67.x, J68.4, J70.1, J70.3
Cardiovascular disease	410.x, 411.x, 412.x, 413.x, 414.x, 427.x, 428.x	I20.x, I21.x, I22.x, I23.x, I25.x, I46.x, I47.x, I48.x, I49.x, I50.x
Cerebrovascular disease	362.34, 430.x–438.x	H34.0x, G45.x, G46.x, I60.x - I69.x
Peripheral vascular disease	093.0, 440.x, 441.x, 443.1, 443.2x, 443.8x, 443.9, 447.1, 557.1, 557.9, V43.4	A52.01, I70.x, I71.x, I73.1, I73.8x, I73.9, I77.1, I79.x, K55.1, K55.8, K55.9, Z95.82x
Diabetes	250.x	E10.x, E11.x, E13.x
Liver disease	070.22, 070.23, 070.32, 070.33, 070.44, 070.54, 070.6, 070.9, 456.0, 456.1, 456.2x, 570.x, 571.x, 572.2, 572.3, 572.4, 572.8, 573.3, 573.4, 573.8, 573.9, V42.7	B18.x, K70.0, K70.1x, K70.2, K70.3x, K70.4x, K70.9, K71.1x, K71.3, K71.4, K71.5x, K71.7, K72.1x, K72.9x, K73.x, K74.x, K76.x, Z94.4, I85.0x, I86.4
Renal disease	403.x, 404.x, 582.x, 583.0 - 583.7, 585.x, 586.x, 588.0, V42.0, V45.11, V56.x	I12.x, I13.x, N03.x, N05.2-N05.7, N18.x, N19.x, N25.0, Z94.0, Z99.2, Z49.x
Obesity	278.0x	E66.x
Smoking	305.1	Z72.0, F17.x
Wheelchair use	V46.3	Z99.3
Hemiplegia or paraplegia	334.1, 342.x, 343.x, 344.0x–344.6x, 344.9	G04.1, G11.4, G80.1, G80.2, G81.x, G82.x, G83.0x - G83.4x, G83.9
Dysphagia	787.2x	R13.1x

Supplemental Table 2. Comparisons of COVID-19 outcomes in excluding MG patients with co-existent RA, SLE or MS

	No. (%)	Crude RR (95% CI)	p-value	Adjusted RR* in reference to None (95% CI)	p-value	Adjusted RR* in reference to MG (95% CI)	p-value
**Hospitalization**							
None	57,613 (14.0)	1.00 (Reference)		1.00 (Reference)		0.79 (0.68 - 0.90)	0.001
MG	121 (37.5)	2.67 (2.32 - 3.07)	<0.001	1.26 (1.10 - 1.46)	0.001	1.00 (Reference)	
RA	1,903 (25.8)	1.84 (1.77 - 1.92)	<0.001	1.00 (0.96 - 1.04)	0.97	0.79 (0.69 - 0.91)	0.001
SLE	315 (23.8)	1.70 (1.54 - 1.87)	<0.001	1.20 (1.10 - 1.32)	<0.001	0.94 (0.80 - 1.11)	0.49
MS	358 (23.6)	1.68 (1.53 - 1.84)	<0.001	1.39 (1.28 - 1.52)	<0.001	1.09 (0.93 - 1.28)	0.29
**ICU**							
None	13,561 (3.3)	1.00 (Reference)		1.00 (Reference)		0.64 (0.48 - 0.85)	0.002
MG	41 (12.7)	3.84 (2.89 - 5.12)	<0.001	1.53 (1.15 - 2.03)	0.004	1.00 (Reference)	
RA	503 (6.8)	2.07 (1.90 - 2.25)	<0.001	1.00 (0.92 - 1.09)	0.95	0.64 (0.48 - 0.86)	0.003
SLE	79 (6.0)	1.81 (1.46 - 2.24)	<0.001	1.19 (0.96 - 1.46)	0.11	0.76 (0.54 - 1.08)	0.12
MS	92 (6.1)	1.83 (1.50 - 2.24)	<0.001	1.45 (1.20 - 1.76)	<0.001	0.92 (0.65 - 1.29)	0.64
**Ventilator**							
None	4,892 (1.2)	1.00 (Reference)		1.00 (Reference)		0.92 (0.51 - 1.64)	0.77
MG	11 (3.4)	2.86 (1.60 - 5.11)	<0.001	1.04 (0.58 - 1.88)	0.90	1.00 (Reference)	
RA	161 (2.2)	1.84 (1.57 - 2.14)	<0.001	0.91 (0.78 - 1.06)	0.23	0.84 (0.46 - 1.53)	0.57
SLE	32 (2.4)	2.03 (1.44 - 2.86)	<0.001	1.33 (0.94 - 1.88)	0.10	1.19 (0.61 - 2.33)	0.61
MS	33 (2.2)	1.82 (1.30 - 2.56)	0.001	1.32 (0.95 - 1.83)	0.10	1.20 (0.61 - 2.33)	0.60
**Death**							
None	12,211 (3.0)	1.00 (Reference)		1.00 (Reference)		0.86 (0.64 - 1.16)	0.33
MG	36 (11.1)	3.75 (2.75 - 5.10)	<0.001	1.16 (0.86 - 1.55)	0.33	1.00 (Reference)	
RA	518 (7.0)	2.37 (2.17 - 2.57)	<0.001	1.02 (0.94 - 1.10)	0.72	0.88 (0.65 - 1.19)	0.41
SLE	75 (5.7)	1.91 (1.53 - 2.38)	<0.001	1.61 (1.31 - 1.99)	<0.001	1.38 (0.97 - 1.98)	0.076
MS	79 (5.2)	1.75 (1.41 - 2.17)	<0.001	1.54 (1.26 - 1.87)	<0.001	1.33 (0.94 - 1.90)	0.11

Abbreviations: MG, myasthenia gravis; RA, rheumatoid arthritis; SLE, systemic lupus erythematosus; MS, multiple sclerosis; RR, risk ratio.

*Adjusted for age, sex, race/ethnicity, region, COVID month, chronic pulmonary disease, cardiovascular disease, cerebrovascular disease, peripheral vascular disease, diabetes, liver disease, renal disease, obesity, and smoking.
